# Burden of Valvular Heart Diseases in a Racially and Ethnically Diverse Population: The Bronx‐Valve Registry

**DOI:** 10.1161/JAHA.124.035378

**Published:** 2025-01-16

**Authors:** Andrea Scotti, Matteo Sturla, Julio Echarte‐Morales, Edwin C. Ho, Antonella Millin, Augustin Coisne, Sebastian Ludwig, Pier Pasquale Leone, Joel Rosiene, Juan F. Granada, Ythan Goldberg, Min Pu, Ulrich P. Jorde, Leandro Slipczuk, Carlos J Rodriguez, Mario J. Garcia, Azeem Latib

**Affiliations:** ^1^ Montefiore‐Einstein Center for Heart and Vascular Care, Montefiore Medical Center Albert Einstein College of Medicine Bronx NY USA; ^2^ Cardiovascular Research Foundation New York NY USA

**Keywords:** diversity, ethnicity, prevalence, race, valvular heart disease, Disparities, Health Equity, Valvular Heart Disease

## Abstract

**Background:**

Valvular heart disease (VHD) is a major focus of cardiovascular medicine, but limited data are available for racial and ethnic minorities. The aim was to assess the burden and clinical correlates of VHD in a highly diverse area of the United States.

**Methods and Results:**

Individuals with echocardiographic diagnosis of native VHD between January 2010 and December 2019 at a quaternary care health system of the Bronx (New York, USA) were included. Prevalence and correlates of VHD were assessed per each racial and ethnic group. From a total of 330 570 adult echocardiograms, 80 584 individuals were diagnosed with VHD and included in the final study population. Stratified by race and ethnicity, 38.0%, 23.2%, 2.1%, and 36.7% were non‐Hispanic Black, non‐Hispanic White, Asian, and Hispanic, respectively. The mean age was 67.7±16.3 years, with non‐Hispanic Black, non‐Hispanic Asian, and Hispanic individuals being younger and having a higher burden of comorbidities. The prevalence of VHD increased with age, irrespective of race or ethnicity. In people aged ≥75 years, tricuspid and mitral regurgitation were the most prevalent VHD (21.1% and 16.1%, respectively). Non‐Hispanic White individuals more frequently had tricuspid regurgitation, mitral regurgitation, and multiple VHDs, but among those aged <65 years, these were more frequent in non‐Hispanic Black individuals.

**Conclusions:**

Our Bronx‐Valve Registry illustrates that the burden of VHD is high, increases with age, and varies among racial and ethnic groups. When diagnosed with VHD, non‐Hispanic Black, non‐Hispanic Asian, and Hispanic individuals are younger and with a higher burden of comorbidities. Appropriate resources and strategies need to be implemented to minimize racial and ethnic disparities and promote equity in VHD diagnosis and cardiovascular risk factor management.

**Registration:**

URL: https://clinicaltrials.gov. Unique Identifier: NCT05453526.

## Graphical abstract

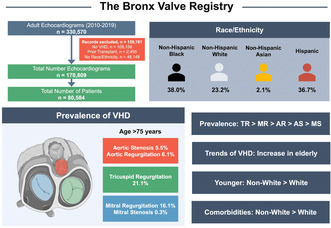

**Nonstandard Abbreviations and Acronyms**
AS: aortic stenosis
MR: mitral regurgitation
SMD: standardized mean difference
TR: tricuspid regurgitation
VHD: valvular heart disease

**Clinical Perspective**

**What Is New?**

- This study scrutinizes the burden and clinical correlates of valvular heart disease in diverse racial and ethnic groups in the Bronx between 2010 and 2019, revealing notable disparities.
- With 80 584 individuals diagnosed from 330 570 echocardiograms, valvular heart disease prevalence was significantly high and increased with age across all racial and ethnic groups.
- Non‐Hispanic Black, non‐Hispanic Asian, and Hispanic individuals were younger with a higher burden of comorbidities. Critical racial and ethnic disparities were evident in valvular heart disease types, specifically for tricuspid and mitral regurgitation.

**What Are the Clinical Implications?**

- The disparities highlight a need for strategically tailored resources and strategies to ensure equitable diagnosis and management across all communities, thereby reducing racial and ethnic discrepancies in valvular heart disease and cardiovascular management.

Cardiovascular disease is the leading cause of death in the general US population.^1^, ^2^ Although the burden of valvular heart disease (VHD) is increasing because of improved survival and the aging population, patients can benefit from improved and more accessible imaging modalities and novel minimally invasive therapies. This translates into early detection and improved outcomes after VHD diagnosis and treatment. However, medical knowledge and technological developments related to VHD might not be generalizable to all patient backgrounds due to the lack of diversity in the scientific literature. Historically, clinical trials testing new treatment modalities have lacked equitable inclusion of people from racial and ethnic minority groups.^3^, ^4^, ^5^ Similarly, epidemiological studies on VHD have focused on specific patient subsets with few direct comparisons across diverse racial and ethnic backgrounds.^6^, ^7^, ^8^ As a result, patients who are racial and ethnic minorities have been found to receive suboptimal care as expressed by various metrics in the field of cardiology.^9^ The lack of diversity in scientific research is an obstacle to reducing disparities and advancing health care equity across different population subgroups.

Bronx County (New York, USA) is a unique urban setting considered by the US Census as the most diverse area in the country, being the only borough in all of New York City predominantly populated by non‐Hispanic Black and Caribbean Hispanic communities.^10^, ^11^ These demographic characteristics make this county ideal to assess how the prevalence and the clinical correlates of VHD might vary among diverse populations. On this background, the objective of the Bronx‐Valve Registry (NCT05453526) was to assess the burden of VHD and explore its clinical correlates in this highly diverse area of the United States.

## Methods

### Study Design

The Bronx‐Valve Registry is an all‐comers registry of individuals with echocardiographic diagnosis of VHD made at the Montefiore Health System in Bronx County. Data from all inpatient and outpatient transthoracic/transesophageal echocardiographic evaluations at Montefiore Medical Center hospital network (Bronx County) were evaluated according to prespecified inclusion/exclusion criteria. The study complied with the Declaration of Helsinki and was approved by the Institutional Review Board of Montefiore Medical Center/Albert Einstein College of Medicine. Informed consent was waived. The study followed the Strengthening the Reporting of Observational Studies in Epidemiology reporting guideline. The data that support the findings of this study are available from the corresponding author upon reasonable request.

### Inclusion Criteria and Definitions

All adult people (aged >18 years) with an echocardiographic diagnosis of native VHD were included in this registry. VHDs of interest were aortic stenosis (AS), aortic regurgitation, mitral stenosis, mitral regurgitation (MR), and tricuspid regurgitation (TR). The assessment of valvular stenosis and regurgitation was carried out as recommended by the American Society of Echocardiography.^12^ Grading of VHD severity was further categorized as mild, moderate, or severe. Patients with mild to moderate and moderate to severe grades of VHD were classified as having moderate and severe grades, respectively. This classification approach was uniformly applied across all VHD subtypes. Native VHD was defined as the absence of previous cardiac valvular surgery on the index valve. Single native VHD was defined as a mild or greater stenotic or regurgitant lesion on a single valve. When analyzing multivalve disease, combined stenotic and regurgitant lesions on the same valve (eg, AS and aortic regurgitation on the aortic valve) were considered as single‐valve disease, maintaining the focus on the number of affected valves rather than the type of lesions. Double and triple VHD were thus defined as the presence of moderate or greater lesions on any 2 or all 3 native heart valves of interest, respectively. Any VHD considered patients with a stenotic or regurgitant grade of moderate or greater affecting at least 1 of the cardiac valves of interest (aortic, mitral, or tricuspid valve). Patients with a history of prior heart transplantation were excluded from the analysis. The total of individuals with and without native VHD (ie, all patients receiving a clinically indicated echocardiogram in the Montefiore Health System) was used to estimate the prevalence of VHD. Patients with multiple VHD and previous valvular interventions were included in the analyses, considering their native lesions only, if there were any.

### Definition of Race and Ethnicity

Race and ethnicity were self‐identified at the time of initial registration for care. A single categorization was used to combine race and ethnicity into 4 mutually exclusive groups: non‐Hispanic Black (“Black”), non‐Hispanic White (“White”), non‐Hispanic Asian (“Asian”), and Hispanic.^13^, ^14^ The Hispanic category was first defined on the basis of patient self‐identification. Then, non‐Hispanic individuals were categorized on the basis of the specified race. Being Hispanic is an ethnicity, not a race, and the self‐reporting of race among individuals of Hispanic ethnicity can be complex and inaccurate due to several cultural, social, and contextual factors.^2^, ^15^ This is not surprising since race is a social construct and the social context of race in Latin America is not an absolute equivalent to that in the United States. Participants lacking explicit racial or ethnic identifiers, either because they did not provide them or they were not available, were excluded from the analysis.

### Data Collection and Study End Points

Data were retrospectively collected over a 10‐year period, from January 1, 2010, to December 31, 2019. All transthoracic/transesophageal echocardiographic reports were retrieved and screened for the presence of VHD diagnosis. The following variables were collected: patient demographics, comorbidities, medical history, previous cardiac surgery/intervention, and echocardiographic data.

The primary end point was the prevalence of VHD according to race and ethnicity. Patient characteristics and primary end point were compared using data from the last available echocardiography. VHD prevalence was further explored according to the following age categories: 18 to 44 years, 45 to 54 years, 55 to 64 years, 65 to 74 years, and ≥75 years.

### Statistical Analysis

Continuous variables were reported as mean±SD or median (interquartile range). Categorical variables were reported as absolute and relative frequencies. Patient characteristics and VHD prevalence among groups were compared by generating a standardized difference. Temporal trends in the prevalence of VHD from 2010 to 2019 were assessed using data from the last available echocardiography of each patient per every 2 years (numerator) and the total number of exams performed in the same period (denominator). The Cochran–Armitage test was used to assess linear trends in proportions of VHD prevalence over time. A *P* value of <0.05 was considered indicative of a statistically significant trend. For all analyses, standardized mean difference (SMD) was determined to be clinically significant with a value of ≥10%. Statistical analyses were performed using R version 4.0.2 (R Foundation for Statistical Computing, Vienna, Austria).

## Results

**Figure 1:**
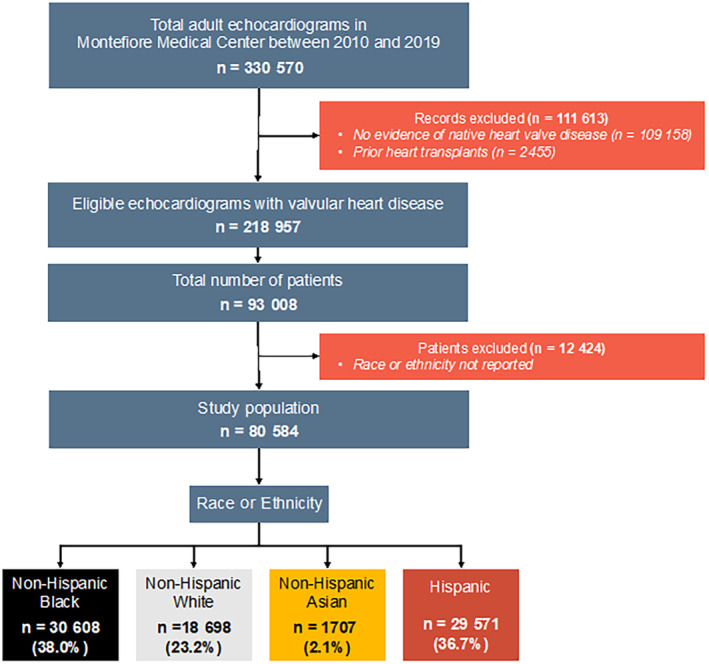
Study flowchart. The flowchart describes the study inclusion and exclusion criteria applied to 330 570 clinically indicated adult echocardiograms performed in the Montefiore Medical Center.

Between January 2010 and December 2019, there were 330 570 echocardiograms performed in the Montefiore Health System (Figure 1). After applying prespecified exclusion criteria, a total of 218 957 echocardiograms reported a diagnosis of VHD in at least 1 native valve. After excluding individuals with race and ethnicity not reported (n=12 424), the final study population consisted of 80 584 individuals. Stratified by race and ethnicity, 38.0%, 23.2%, and 2.1%, were Black, White, and Asian, respectively. Self‐identified Hispanics represented 36.7% of the study population.

### Patient Characteristics

The main baseline characteristics of the study population are presented in Table 1. The mean age was 67.7±16.3 years, with non‐Hispanic Black, non‐Hispanic Asian, and Hispanic individuals being younger. The overall prevalence of hypertension, coronary artery disease, and hyperlipidemia was 53.0%, 24.5%, and 35.8%, respectively, with the non‐Hispanic Black, non‐Hispanic Asian, and Hispanic cohort generally having a higher burden of comorbidities. Compared with White individuals, Black individuals were more likely to have diabetes (27.3% versus 15.0%; SMD, 35.9%) and hypertension (56.1% versus 44.5%; SMD, 23.4%), were more frequently smokers (21.0% versus 11.2%; SMD, 26.8%), and more frequently had chronic kidney disease (33.7% versus 24.3%; SMD, 20.9%). Rather, White individuals more frequently had atrial fibrillation compared with Black individuals (19.8% versus 10.7%; SMD, 28.2%) and were more likely affected by coronary artery disease (28.4% versus 20.3%; SMD, 19.1%). Considering the Hispanic population, they were more likely to be smokers (20.4%), have diabetes (29.5%), and have chronic obstructive lung disease (19.4%). Stratifying the study population per severity of VHD (ie, mild VHD versus ≥moderate VHD), individuals with more advanced stages of VHD were older and had more frequent coronary artery disease and atrial fibrillation, with no significant differences in the remaining comorbidities (Table S1).

**Table 1:** Baseline Clinical Characteristics by Race and Ethnicity

Characteristic	Overall, n=80 584	Non‐Hispanic Black, n=30 608 (38.0%)	Non‐Hispanic White, n=18 698 (23.2%)	Non‐Hispanic Asian, n=1707 (2.1%)	Hispanic, n=29 571 (36.7%)	SMD* (%)
Age, y	67.7 ± 16.3	65.4 ± 16.3	75.0 ± 14.3	65.8 ± 14.5	65.7 ± 16.3	64.1
Female sex	47 501 (59.0)	19 044 (62.2)	9682 (51.8)	852 (49.9)	17 923 (60.6)	25.0
Body surface area	1.8 ± 0.3	1.9 ± 0.3	1.8 ± 0.3	1.7 ± 0.2	1.8 ± 0.2	78.6
Body mass index	28.7 ± 7.2	29.3 ± 7.9	27.9 ± 6.7	26.0 ± 5.5	28.8 ± 6.7	47.4
Heart rate, bpm	79.2 ± 20.6	79.9 ± 21.0	83.9 ± 21.5	77.4 ± 16.4	77.8 ± 20.2	34.1
Systolic blood pressure, mm Hg	131.3 ± 21.6	133.2 ± 22.1	129.5 ± 21.7	130.3 ± 21.5	130.2 ± 20.9	17.0
Diastolic blood pressure, mm Hg	71.2 ± 13.7	73.3 ± 14.0	68.9 ± 13.4	70.1 ± 13.5	70.3 ± 13.3	32.3
Diabetes	20 405 (25.3)	8363 (27.3)	2812 (15.0)	508 (29.8)	8722 (29.5)	35.9
Hypertension	42 737 (53.0)	17 177 (56.1)	8322 (44.5)	812 (47.6)	16 426 (55.6)	23.4
Smoker	14 749 (18.3)	6418 (21.0)	2098 (11.2)	210 (12.3)	6023 (20.4)	26.8
Stroke	5690 (7.1)	2462 (8.0)	869 (4.7)	112 (6.6)	2247 (7.6)	14.0
Peripheral vascular disease	10 367 (12.9)	3974 (13.0)	2002 (10.7)	198 (11.6)	4193 (14.2)	10.5
Chronic obstructive pulmonary disease	13 386 (16.6)	5286 (17.3)	2140 (11.5)	221 (13.0)	5739 (19.4)	22.2
Coronary artery disease	19 724 (24.5)	6206 (20.3)	5315 (28.4)	466 (27.3)	7737 (26.2)	19.1
Atrial fibrillation	10 653 (13.2)	3261 (10.7)	3707 (19.8)	169 (9.9)	3516 (11.9)	28.2
Previous myocardial infarction	1943 (2.4)	622 (2.0)	433 (2.3)	69 (4.0)	819 (2.8)	11.7
Coronary artery bypass grafting	1616 (2.0)	370 (1.2)	399 (2.1)	85 (5.0)	762 (2.6)	21.9
Hyperlipidemia	28 870 (35.8)	10 853 (35.5)	6147 (32.9)	611 (35.8)	11 259 (38.1)	10.9
Chronic kidney disease	24 951 (31.0)	10 327 (33.7)	4547 (24.3)	561 (32.9)	9516 (32.2)	20.9
Rheumatic heart disease	3866 (4.8)	1199 (3.9)	1307 (7.0)	80 (4.7)	1280 (4.3)	13.6

Values are mean±SD or n (%).SMD indicates standardized mean difference.

* The SMD is a measure of effect size and is calculated as the difference in the mean or proportion between 2 groups divided by the SD of that difference. A standardized difference of >10 percentage points indicates a clinically meaningful difference. The largest SMD calculated from pairwise comparisons among the 4 racial and ethnic groups (non‐Hispanic Black, non‐Hispanic White, non‐Hispanic Asian, and Hispanic) is reported.

### Prevalence of VHD by Race and Ethnicity

The prevalence of VHD in each racial and ethnic group is reported in Table 2. Overall, moderate or greater VHD affecting any valve was present in 35.2% of the final study population, with White individuals being the most affected (41.2%), followed by Black (35.5%), Hispanic (31.3%), and Asian individuals (30.8%; SMD, 21.7%). Similarly, severe VHD was present in 8.7% of the study population, with 11.0% of White individuals, 8.7% of Black individuals, 7.4% of Hispanic individuals, and 5.8% of Asian individuals affected. TR was the most prevalent VHD, followed by MR, both in the overall study population (73.4% and 59.1%, respectively) and in each racial and ethnic group.

**Table 2:** Distribution of Valvular Heart Disease by Race and Ethnicity

Variable	Overall, n=80 584	Non‐Hispanic Black, n=30 608 (38.0%)	Non‐Hispanic White, n=18 698 (23.2%)	Non‐Hispanic Asian, n=1707 (2.1%)	Hispanic, n=29 571 (36.7%)	SMD* ^,^ † (%)
Aortic stenosis						24.6
Mild	2470 (3.1)	656 (2.1)	872 (4.7)	52 (3.1)	890 (3.0)	
Moderate	1772 (2.2)	425 (1.4)	728 (3.9)	25 (1.5)	594 (2.0)	
Severe	1389 (1.7)	307 (1.0)	706 (3.8)	15 (0.9)	361 (1.2)	
Aortic regurgitation						8.7
Mild	16 831 (20.9)	5407 (17.7)	4987 (26.7)	413 (24.2)	6024 (20.4)	
Moderate	4286 (5.3)	1441 (4.7)	1283 (6.9)	115 (6.7)	1447 (4.9)	
Severe	357 (0.4)	154 (0.5)	84 (0.5)	5 (0.3)	114 (0.4)	
Mitral stenosis						
Severe	243 (0.3)	79 (0.3)	77 (0.4)	5 (0.3)	82 (0.3)	2.7
Mitral regurgitation						12.6
Mild	34 311 (42.6)	12 431 (40.6)	8525 (45.6)	783 (45.9)	12 572 (42.5)	
Moderate	11 051 (13.7)	4100 (13.4)	3106 (16.6)	220 (12.9)	3625 (12.3)	
Severe	2243 (2.8)	854 (2.8)	567 (3.0)	34 (2.0)	788 (2.7)	
Tricuspid regurgitation						21.4
Mild	42 417 (52.6)	16 255 (53.1)	9358 (50.1)	868 (50.9)	15 936 (53.9)	
Moderate	13 014 (16.2)	5589 (17.8)	3378 (18.1)	208 (12.2)	3968 (13.4)	
Severe	3700 (4.6)	1668 (5.5)	862 (4.6)	47 (2.8)	1123 (3.8)	
Any moderate or greater VHD	28 335 (35.2)	10 856 (35.5)	7697 (41.2)	526 (30.8)	9256 (31.3)	21.7
Any severe VHD	6983 (8.7)	2655 (8.7)	2055 (11.0)	99 (5.8)	2174 (7.4)	18.8
Double VHD†	6899 (8.6)	2674 (8.7)	2044 (10.9)	111 (6.5)	2070 (7.0)	15.7
Triple VHD†	1108 (1.4)	387 (1.3)	409 (2.2)	16 (0.9)	296 (1.0)	10.1

Values are n (%). SMD indicates standardized mean difference; and VHD, valvular heart disease.

* The SMD is a measure of effect size and is calculated as the difference in the mean or proportion between 2 groups divided by the SD of that difference. A standardized difference of >10 percentage points indicates a clinically meaningful difference. The largest SMD calculated from pairwise comparisons among the 4 racial and ethnic groups (non‐Hispanic Black, non‐Hispanic White, Non‐Hispanic Asian, and Hispanic) is reported.

^†^ SMD calculations were performed considering only patients with a severity grade of moderate or greater.

**Figure 2:**
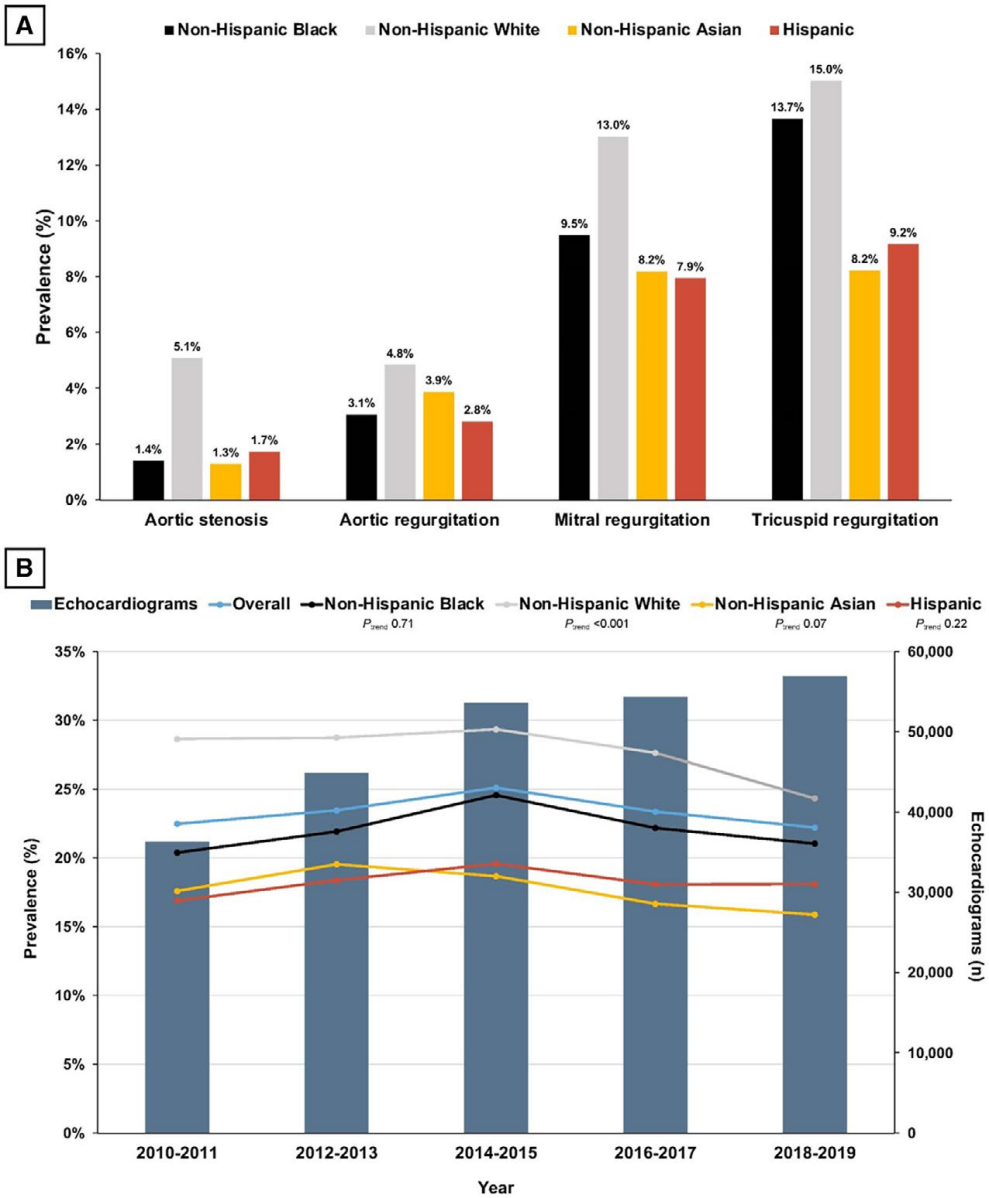
Prevalence of VHD by race and ethnicity. **A**, Overall prevalence of VHD per race and ethnicity from 2010 to 2019. All patients within each racial and ethnic group receiving an echocardiogram were considered as the sample population when determining the prevalence of VHD. B, Number of echocardiograms and prevalence of VHD by year stratified by race and ethnicity. Temporal trend analyses showed a significant reduction in VHD prevalence for White people (Cochrane–Armitage *P*<0.001), a trend for lower VHD in the Asian group (Cochrane–Armitage *P*=0.07), and no differences for Black (Cochrane–Armitage *P*=0.71) and Hispanic (Cochrane–Armitage *P*=0.22) cohorts. All patients receiving an echocardiogram within the defined years were considered as the sample population when determining the prevalence of VHD. VHD was defined as having a severity grade of moderate or greater. VHD indicates valvular heart disease.

A prevalence analysis by race and ethnicity for each VHD with severity equal to or greater than moderate identified White individuals as the most affected, followed by Black individuals (Figure 2A). White individuals were more likely to have moderate or greater AS than Black or Asian individuals. MR was more frequent in White compared with Asian and Black individuals. Moderate or greater TR was more often diagnosed in Black and White cohorts compared with Asian and Hispanic cohorts. Double VHD was present in 8.6% of the overall study population, with a higher proportion in White (10.9%) compared with Black (8.7%; SMD, 7.4%), Hispanic (7.4%, SMD, 13.8%), and Asian individuals (6.5%; SMD, 15.7%). Severe mitral stenosis was the least frequent valve disease in the entire population (0.3%) and in each of the races and ethnicities studied.

### Prevalence of VHD Over Time

There was an increase in the absolute number of clinically indicated echocardiograms performed during the study period (Figure 2B; Figure S1). Temporal trend analyses on the prevalence of moderate or greater VHD showed different findings according to race and ethnicity. While White people had a significant reduction in the prevalence of VHD over time (Cochrane–Armitage *P*<0.001) with a trend for lower VHD in the Asian group (Cochrane–Armitage *P*=0.07), no differences were observed for Black (Cochrane–Armitage *P*=0.71) and Hispanic (Cochrane‐Armitage *P*=0.22) cohorts. Temporal trends of each VHD are analyzed separately and illustrated in Figures S2 and S3.

### Prevalence of VHD by Age

**Figure 3:**
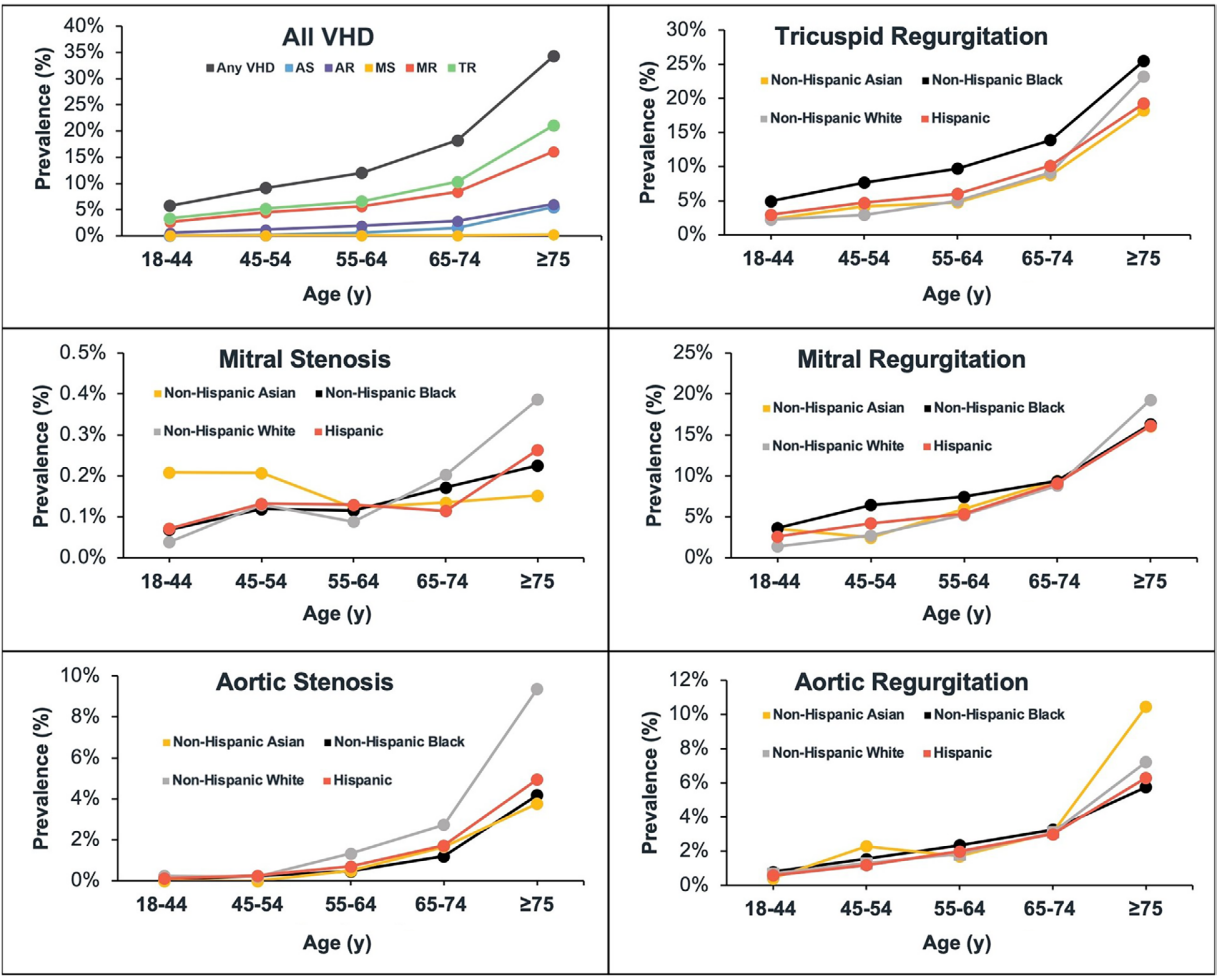
Prevalence of VHD by age. VHD was defined as having a grade of moderate severity or greater. All patients within each age group receiving an echocardiogram were considered as the sample population when determining the prevalence of VHD. AS indicates aortic stenosis; AR, aortic regurgitation; MR, mitral regurgitation; MS, mitral stenosis; TR, tricuspid regurgitation; and VHD, valvular heart disease.

The proportion of individuals with VHD affecting any valve represented in each age group increased with age, with those aged >75 years making up 38.0% of the study population (Table S2). The age‐adjusted prevalence of moderate or severe VHD for each valve disease showed an exponential increase from age 55 years, reaching the maximum in the group aged >75 years (Figure 3; Figures S3 and S4). Notably, AS had a 3‐fold increase in prevalence from the group aged 65 to 74 years to the eldest age group. This sharp rise in detection was consistent across all racial and ethnic subgroups and was most evident in the White cohort. TR, followed by MR, was the most diagnosed VHD in all age subgroups. When stratified by race and ethnicity, young Black individuals had a higher prevalence of TR and MR compared with the other races and Hispanic individuals. When considering double VHD, Black individuals also had the highest prevalence at younger ages (Figure S3).

## Discussion

Our Bronx‐Valve Registry investigated the burden of VHD in one of the most racially and ethnically diverse counties in the United States. In Bronx County, (1) the burden of VHD was high among a predominantly minority population, increased with age, and differed among racial and ethnic groups, with the White population being the most affected; (2) non‐Hispanic Black, non‐Hispanic Asian, and Hispanic individuals with a diagnosis of VHD were younger and with a higher burden of comorbidities; and (3) TR followed by MR were the most common VHDs.

The burden of VHD diagnosed in Bronx County was higher than previous reports coming from Olmsted County. Examining individuals aged ≥75 years receiving an echocardiography, the following prevalence of moderate or greater VHD was observed in Bronx and Olmsted counties: AS (3.9% versus 8.0%), aortic regurgitation (5.7% versus 3.0%), MR (16.5% versus12.4%), and TR (20.8% versus 10.6%).^16^, ^17^ The higher prevalence of VHD in Bronx County might be explained by an aging population over the 20‐year difference in the study periods (Bronx‐Valve, 2010–2019 versus Olmsted County, 1990–2000) and the lack of diversity in Olmsted County, where non‐Hispanic White people represented >90% of the population and non‐Hispanic Black individuals only 2.7%. Except for mitral stenosis, all the VHDs showed a continuous and expected increase in prevalence with age. Furthermore, while other studies have investigated the burden of VHD, they have predominantly focused on a single type of VHD, such as aortic stenosis, and often did not comprehensively investigate the impact of race and ethnicity on disease prevalence and severity.^18^, ^19^, ^20^, ^21^ Investigating the burden of VHD by race and ethnicity, White individuals showed the highest prevalence of AS and MR, Asian individuals were diagnosed more commonly with aortic regurgitation, and significant TR was more prevalent in Black and White individuals. Interestingly, combined VHDs were observed in 8.6% (double VHD) and 1.4% (triple VHD) of people, with White individuals having the highest prevalence at an older age and Black individuals being more diagnosed at a younger age. As observed in the Bronx‐Valve Registry, differences in VHD by race and ethnicity exist and should be acknowledged when designing clinical trials and selecting the participating centers to promote diversity.^4^, ^5^

In the United States, almost 50% of the population is represented by racial and ethnic minorities (non‐Hispanic Black, non‐Hispanic Asian, and Hispanic race or Hispanic/Latino ethnicity).^10^ However, there is no systematic assessment of VHD prevalence in these populations. Similarly, their representation in clinical trials is extremely limited. In 2020, 32 000 individuals participated in clinical trials on novel drugs approved by the Food and Drug Administration, and 75% of them were White individuals.^22^ When it comes to VHD trials, patients' race and ethnicity is not even detailed or reported to be as low as 8.7% for the non‐Hispanic Black, non‐Hispanic Asian, and Hispanic race or ethnic group.^23^ The lack of diversity in clinical trials raises serious concerns on the generalizability of therapies proven to be effective in a White‐prevalent population. As shown in this registry on VHD, there are significant differences according to race and ethnicity in terms of patient characteristics. Non‐Hispanic Black, non‐Hispanic Asian, and Hispanic populations are much younger and have a higher burden of comorbidities, including diabetes, hypertension, and chronic kidney disease, compared with the White group. Similar differences based on race and ethnicity can be observed in non‐VHD populations affected by hyperlipidemia, coronary artery disease, and atrial fibrillation.^24^, ^25^, ^26^ The underrepresentation of racial and ethnic minorities is in stark contrast with their enhanced risk profile, which identifies them as an important target for preventive care and life‐saving therapies. Also, guideline recommendations on VHD provide indications for treatment (eg, surgical versus transcatheter approach) according to the patient's age, and these cutoffs might not be valid among racial and ethnic groups with different risk–benefit profiles.^27^, ^28^

In the Bronx‐Valve Registry, TR was the most prevalent VHD, affecting about 1 of 4 individuals aged >75 years, considering VHD moderate or greater. The “indolent” course of TR and the misperception of tricuspid valve disease as a mere bystander led TR to be long forgotten and not even reported in historical community studies.^16^ Increasing evidence suggests that TR is common and independently associated with adverse clinical outcomes, including excess death.^17^, ^29^ However, surgery for isolated TR is seldom performed because of significant in‐hospital death and postoperative complication rates.^30^ Preliminary findings from a multicenter registry and a randomized clinical trial suggest that transcatheter interventions are safe and effective in providing a significant clinical benefit in patients with TR.^31^, ^32^, ^33^ Currently, no transcatheter devices for TR are commercially approved in the United States, but several trials are underway. Until recently, no transcatheter devices for TR were commercially available in the United States. However, with the recent FDA approval of 2 transcatheter devices for TV repair and replacement, significant strides have been made in addressing the previously unmet need in TR treatment.

VHD represents a growing and serious public health problem, particularly in resource‐poor countries; early VHD detection and treatment can significantly improve life expectancy and should be universally accessible. In this light, we envision our data supporting more targeted public health interventions, including the development and implementation of widespread screening programs specifically designed to identify VHD early in populations at higher risk due to socioeconomic or racial factors. Moreover, improving access to health care in underserved communities stands as a fundamental objective. Such initiatives could significantly benefit from the integration of implementation science, ensuring that interventions are not only evidence based but also culturally tailored and scalable to effectively reach and impact those most at risk. Acknowledging the importance of VHD epidemiology is crucial to promote scientific progress, advancing clinical practice, and formulating correct health policies. Encouraging efforts are being made by authorities (Food and Drug Administration) to encourage diverse participation in clinical trials.^34^ Similarly, medical journals are fostering clarity and transparency in patient demographic characterization with the goal of recruiting appropriately representative participants in studies.^4^ Promoting diversity, equity, and inclusion is not just social justice; it will address a genuine clinical need and ultimately improve the quality of health care.

### Study Limitations

Considering the large, investigated population, echocardiography was the only available method to estimate the prevalence of VHD, and corroboration by other tests was not feasible. All inpatient and outpatient echocardiographic evaluations performed at Montefiore Medical Center, which serves Bronx County, were included in this registry. Our study was limited by race and ethnicity collection by hospital report, which may result in misclassification. The proportion of reports excluded due to lack of reported data on race and ethnicity is in line with previously published epidemiological data related to cardiovascular disease and remains an inherent limitation of population‐based studies.^35^, ^36^ This underscores the need for improved data collection practices in future research, particularly in the gathering of comprehensive racial and ethnic information, to enhance the understanding and management of cardiovascular diseases in diverse populations. Although these data report the prevalence of diagnosed VHD in the community of the Bronx, the true burden of VHD might be underestimated by undiagnosed VHD and possibly biased by access to health care. As an all‐comer registry, the sensitivity in detecting VHD might have varied according to the primary focus of the echocardiographic study (ie, higher when performed for VHD assessment). Also, additional studies might have been required to better evaluate the severity of VHD (ie, transesophageal echocardiography). For this reason, the most recent echocardiographic report of each patient was used for the analyses.

### Conclusions

The Bronx‐Valve Registry illustrates that the burden of VHD is high in a predominantly minority population, increases with age, and differs among racial and ethnic groups. When diagnosed with VHD, non‐Hispanic Black, non‐Hispanic Asian, and Hispanic people are younger and with a higher burden of comorbidities. Appropriate resources and strategies need to be implemented to minimize racial and ethnic disparities and promote equity in VHD diagnosis, cardiovascular risk factor management, and access to health care.

## Sources of Funding

None.

## Disclosures

Dr Scotti has served as a consultant for Edwards Lifesciences and NeoChord Inc. Dr Echarte‐Morales was supported by Sociedad Española de Cardiología through a mobility grant (SEC/PRS‐MOV‐INT 22/001). Dr Ludwig has received travel compensation from Edwards LifeSciences, advisory fees from Bayer, and speaker honoraria from Abbott. Dr Latib has served on the advisory board for Medtronic, Abbott Vascular, Boston Scientific, Edwards Lifesciences, Shifamed, NeoChord Inc, V‐dyne, and Philips. The remaining authors have no disclosures to report.

## Supporting information

Tables S1–S2
Figures S1–S4
