# Supplementary material for: Burden of Valvular Heart Diseases in a Racially and Ethnically Diverse Population: The Bronx‐Valve Registry
Source: J Am Heart Assoc. 2025 Jan 16;14(2):e035378. doi: 10.1161/JAHA.124.035378 (PMC12054424; doi:10.1161/JAHA.124.035378)

SUPPLEMENTAL MATERIAL

Table S1. Baseline Characteristics Stratified by Race/Ethnicity and Severity of Valvular Heart Disease.

Characteristic	Overall n=80,584			Black n=30,608 (38.0%)			Hispanic n=29,571 (36.7%)			White n=18,698 (23.2%)			Asian n=1,707 (2.1%)		
	Mild VHD n=52,249	≥Moderate VHD n=28,335	SMD†	Mild VHD n=19,752	≥Moderate VHD n=10,856	SMD†	Mild VHD n=20,315	≥Moderate VHD n=9,256	SMD†	Mild VHD n=11,001	≥Moderate VHD n=7,7697	SMD†	Mild VHD n=1,181	≥Moderate VHD n=526	SMD†
**Age, years**	64.9±16.2	73.0±15.2	51.2	62.9±16.1	70.0±15.7	44.5	63.3±16.2	71.0±15.1	49.5	71.8±14.6	79.7±12.3	58.5	63.7±14.3	70.4±14.0	55.6
**Female**	30,429 (58.2)	17,072 (60.3)	4.1	12,115 (61.3)	6,929 (63.8)	5.1	12,345 (60.8)	5,578 (60.3)	1.0	5,374 (48.9)	4,308 (56.0)	14.3	595 (50.4)	257 (48.9)	3.1
**BSA**	1.87±0.3	1.81±0.3	23.6	1.92±0.3	1.86±0.3	22.3	1.82±0.2	1.76±0.2	23.0	1.87±0.3	1.78±0.3	33.3	1.73±0.2	1.68±0.2	59.4
**BMI**	29.24± 7.2	27.80±7.1	20.1	29.77±7.8	28.32±7.8	18.5	29.24±6.7	28.00±6.6	18.6	28.57± 6.8	26.80±6.5	26.7	26.41±5.4	25.12±5.6	35.2
**Heart Rate, bpm**	77.62±19.8	82.86±22.0	25.0	78.62±20.6	82.77±21.7	19.6	76.38±19.3	81.24±21.8	23.6	80.59±19.4	88.66±23.4	37.6	77.29±17.2	77.70±15.0	18.0
**Systolic BP, mmHg**	131.9±21.2	130.2±22.2	7.7	134.0±21.4	131.9±23.1	9.7	130.5±20.7	129.7±21.4	3.6	130.7±21.6	127.7±21.8	14.1	130.4±22.0	129.8±20.4	1.4
**Diastolic BP, mmHg**	72.0±13.3	70.0±14.3	14.7	74.0±13.5	72.2±14.7	12.6	71.0±12.9	69.0±14.0	15.3	70.0±13.3	67.3±13.4	19.7	71.0±13.5	67.9±13.4	7.7
**Diabetes**	12,997 (24.9)	7,408 (26.1)	2.9	5,270 (26.7)	3,093 (28.5)	4.1	5,650 (27.8)	3,072 (33.2)	11.7	1,718 (15.6)	1,094 (14.2)	3.9	149 (28.3)	359 (30.4)	35.7
**Hypertension**	27,384 (52.4)	15,353 (54.2)	3.6	11,011 (55.7)	6,166 (56.8)	2.1	10,952 (53.9)	5,474 (59.1)	10.6	4,867 (44.2)	3,455 (44.9)	1.3	258 (49.0)	554 (46.9)	5.4
**Smoker**	10,015 (19.2)	4,734 (16.7)	6.4	4,251 (21.5)	2167 (20.0)	3.8	4,274 (21.0)	1,749 (18.9)	5.4	1,346 (12.2)	752 (9.8)	7.9	66 (12.5)	144 (12.2)	0.1
**Stroke**	3,380 (6.5)	2,310 (8.2)	6.5	1,464 (7.4)	998 (9.2)	6.5	1,370 (6.7)	877 (9.5)	10.0	481 (4.4)	388 (5.0)	3.2	47 (8.9)	65 (5.5)	5.2
**Peripheral vascular disease**	6,127 (11.7)	4,240 (15.0)	9.5	2,302 (11.7)	1,672 (15.4)	11.0	2,598 (12.8)	1,595 (17.2)	12.5	1,098 (10.0)	904 (11.7)	5.7	69 (13.1)	129 (10.9)	3.1
**COPD**	8,808 (16.9)	4,578 (16.2)	1.9	3,381 (17.1)	1,905 (17.5)	1.1	3,967 (19.5)	1,772 (19.1)	1.0	1,303 (11.8)	837 (10.9)	3.1	64 (12.2)	157 (13.3)	4.4
**CAD**	11,822 (22.6)	7,902 (27.9)	12.1	3,653 (18.5)	2,553 (23.5)	12.4	4,854 (23.9)	2,883 (31.1)	16.3	3,007 (27.3)	2,308 (30.0)	5.9	158 (30.0)	308 (26.1)	2.8

**Atrial Fibrillation**	4,960 (9.5)	5,693 (20.1)	30.2	1,452 (7.4)	1,809 (16.7)	28.9	1,690 (8.3)	1,826 (19.7)	33.3	1,736 (15.8)	1,971 (25.6)	24.4	87 (16.5)	82 (6.9)	28.1
**Previous MI**	1,133 (2.2)	810 (2.9)	4.4	355 (1.8)	267 (2.5)	4.6	499 (2.5)	320 (3.5)	5.9	234 (2.1)	199 (2.6)	3.0	24 (4.6)	45 (3.8)	9.9
**CABG**	919 (1.8)	697 (2.5)	4.9	208 (1.1)	162 (1.5)	3.9	438 (2.2)	324 (3.5)	8.1	220 (2.0)	179 (2.3)	2.2	32 (6.1)	53 (4.5)	14.1
**Hyperlipidemia**	18,794 (36.0)	10,076 (35.6)	0.9	6,993 (35.4)	3,860 (35.6)	0.3	7,615 (37.5)	3,644 (39.4)	3.9	3,750 (34.1)	2,397 (31.1)	6.3	175 (33.3)	436 (36.9)	5.9
**CKD**	15,298 (29.3)	9,653 (34.1)	10.3	6,177 (31.3)	4,150 (38.2)	14.6	6,071 (29.9)	3,445 (37.2)	15.6	2,670 (24.3)	1,877 (24.4)	0.3	181 (34.4)	380 (32.2)	17.6
**Rheumatic heart disease**	1,670 (3.2)	2,196 (7.8)	20.1	492 (2.5)	707 (6.5)	19.5	535 (2.6)	745 (8.0)	24.3	606 (5.5)	701 (9.1)	13.9	43 (8.2)	37 (3.1)	11.7

Values are mean ± SD, n (%).
† The SMD is a measure of effect size and is calculated as the difference in the mean or proportion between two groups divided by the standard deviation of that difference. A standardized difference of greater than 10 percentage points indicates a clinically meaningful difference. The largest SMD calculated from pairwise comparisons among the four racial groups (Non-Hispanic Black, Non-Hispanic White, Non-Hispanic Asian and Hispanic) is reported.
BP: blood pressure; BSA: body surface area; BMI: body mass index; CABG: coronary artery bypass grafting; CAD: coronary artery disease; CKD: chronic kidney disease; COPD: chronic obstructive pulmonary disease; MI: myocardial infarction; SMD: standardized mean difference.

Table S2. Distribution of Valvular Heart Disease by Age

Variable	Overall<br>n=80,584	18-44 years<br>n=7,227 (9.0%)	45-54 years<br>n=9,110 (11.3%)	55-64 years<br>n=15,345 (19.0%)	65-74 years<br>n=18,257 (22.7%)	≥75 years<br>n=30,645 (38.0%)
**Aortic Stenosis**						
**Mild**	2,470 (3.1)	32 (0.4)	65 (0.7)	257 (1.7)	544 (3.0)	1,572 (5.1)
**Moderate**	1,772 (2.2)	15 (0.2)	31 (0.3)	135 (0.9)	313 (1.7)	1,278 (4.2)
**Severe**	1,389 (1.7)	7 (0.1)	17 (0.2)	75 (0.5)	191 (1.1)	1,099 (3.6)
**Aortic Regurgitation**						
**Mild**	16,831 (20.9)	499 (6.9)	1,021 (11.2)	2,367 (15.4)	3,836 (21.0)	9,108 (29.7)
**Moderate**	4,286 (5.3)	116 (1.6)	259 (2.8)	571 (3.7)	864 (4.7)	2,476 (8.1)
**Severe**	357 (0.4)	32 (0.4)	42 (0.5)	75 (0.5)	73 (0.4)	135 (0.4)
**Mitral Stenosis**						
**Severe**	243 (0.3)	15 (0.2)	28 (0.3)	36 (0.2)	47 (0.3)	117 (0.4)
**Mitral Regurgitation**						
**Mild**	34,311 (42.6)	2,837 (39.3)	3,959 (21.1)	6,858 (44.7)	7,778 (42.6)	12,879 (42.0)
**Moderate**	11,051 (13.7)	534 (7.4)	879 (9.7)	1,579 (10.3)	2,331 (12.8)	5,728 (18.7)
**Severe**	2,243 (2.8)	100 (1.3)	194 (2.1)	333 (2.2)	419 (2.3)	1,197 (3.9)
**Tricuspid Regurgitation**						
**Mild**	42,417 (32.8)	4,636 (64.2)	5,134 (56.4)	8,404 (54.8)	9,609 (52.6)	14,634 (47.8)
**Moderate**	13,014 (10.1)	654 (9.1)	994 (10.9)	1,776 (11.6)	2,669 (14.6)	6,920 (22.6)
**Severe**	3,700 (2.9)	142 (2.0)	250 (2.7)	459 (3.0)	712 (3.9)	2,136 (7.0)
**Double VHD***	6,899 (8.6)	217 (3.0)	433 (4.8)	777 (5.1)	1,258 (6.9)	4,214 (13.8)
**Triple VHD***	1,108 (1.4)	11 (0.2)	27 (0.3)	60 (0.4)	140 (0.8)	870 (2.8)

Values are n (%). *Considering valvular heart diseases with a severity grade ≥ moderate. VHD: valvular heart disease.

Figure S1. Number of Echocardiograms Performed Between 2010 and 2019 Stratified by Race/Ethnicity.

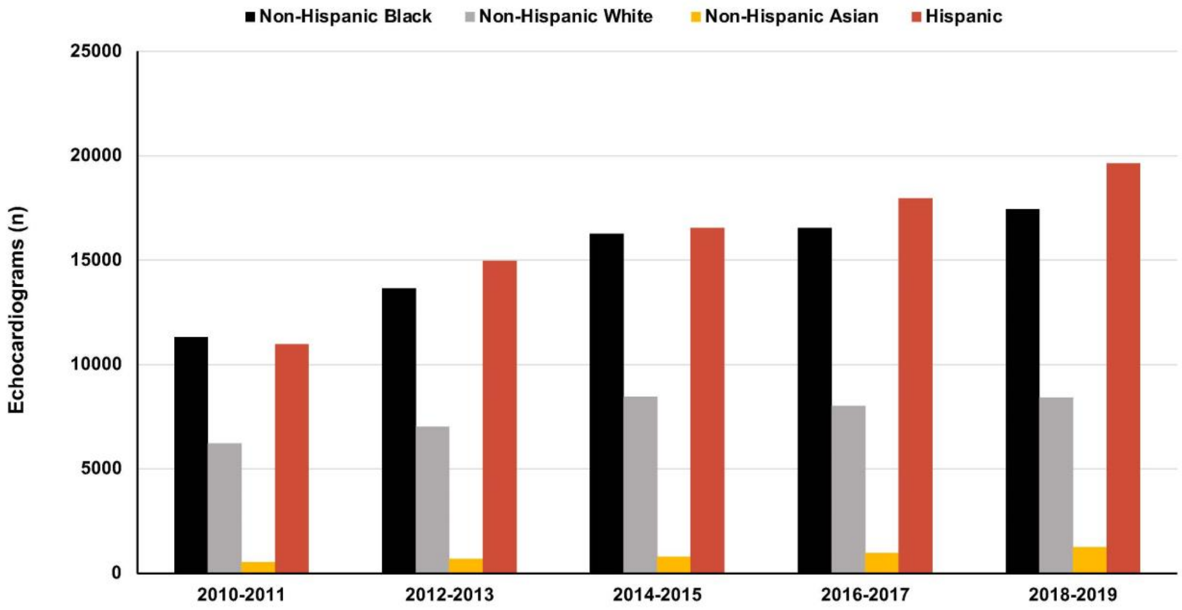

Figure S2. Prevalence of Valvular Heart Disease from 2010 to 2019 Stratified by Race/Ethnicity.

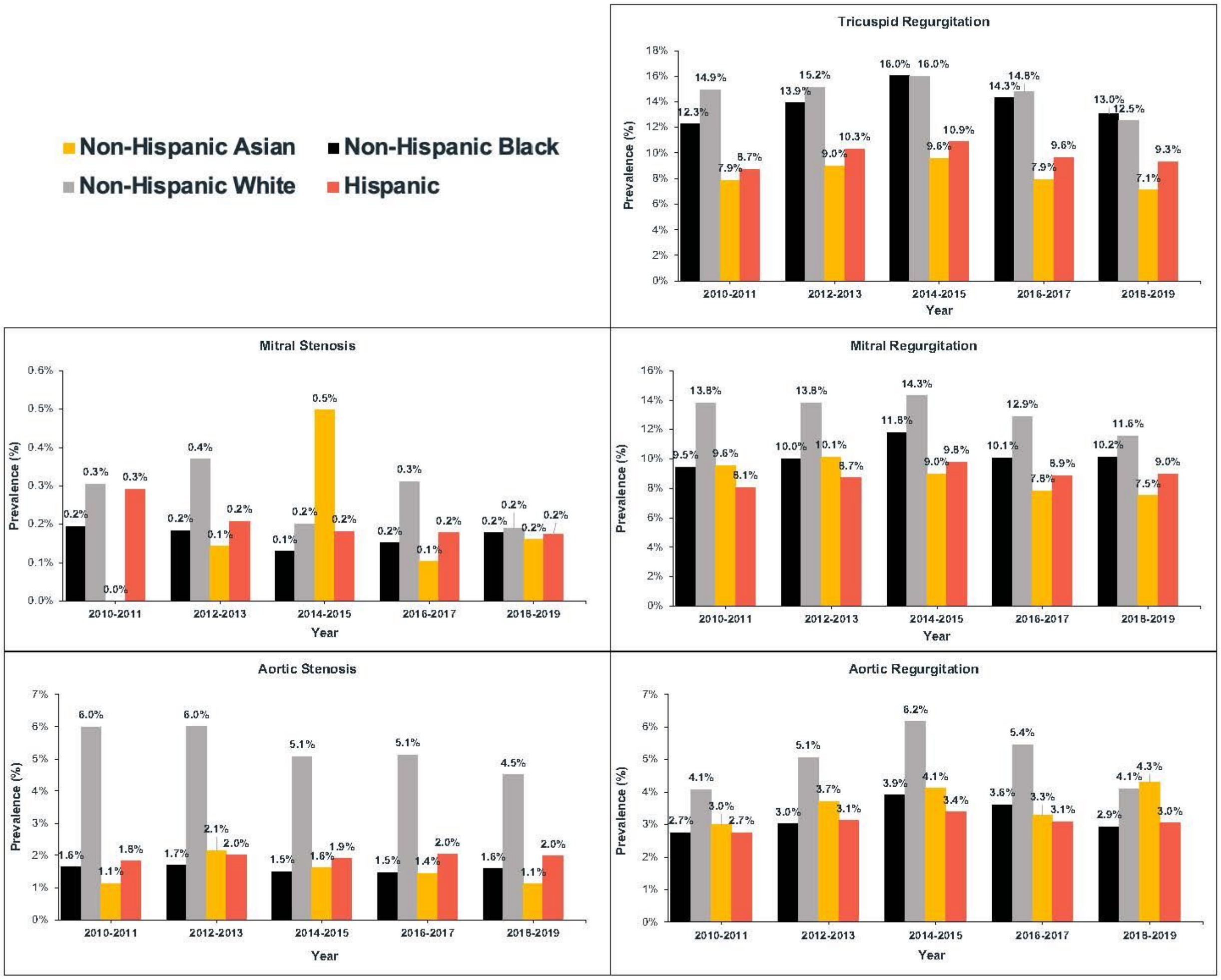

Figure S3. Prevalence of Double and Triple Valve Disease According to Age (Top Panels) and Year (Bottom Panels) Stratified by Race/Ethnicity.

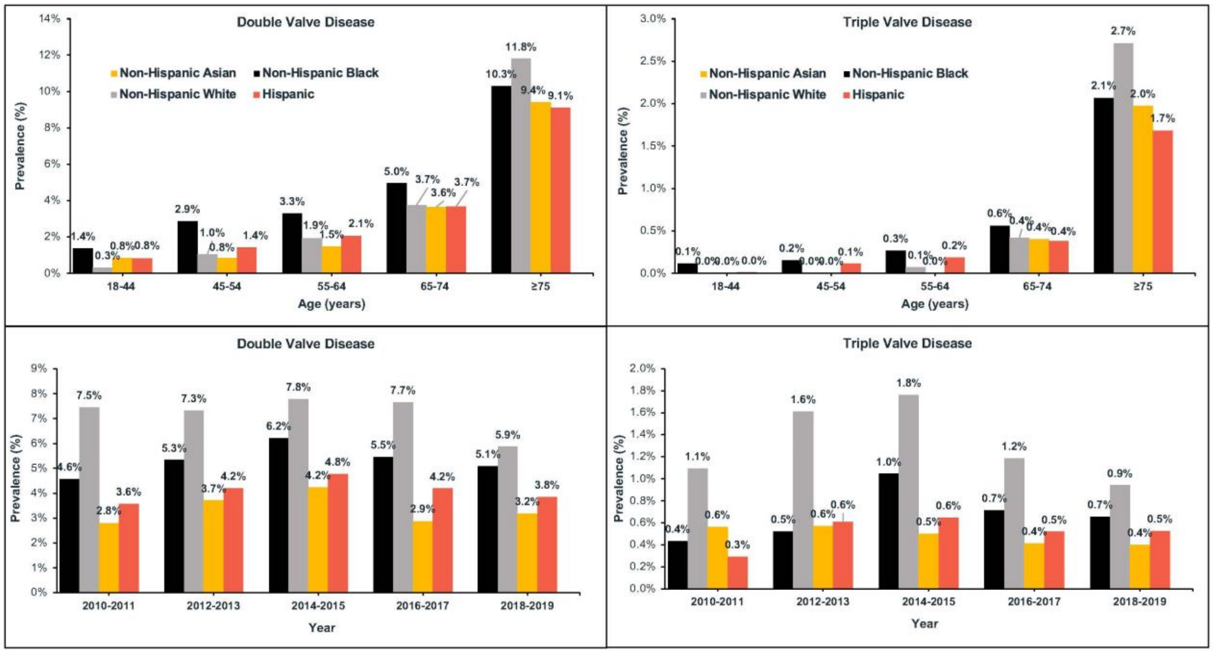

Figure S4. Prevalence of Valvular Heart Disease by Age.

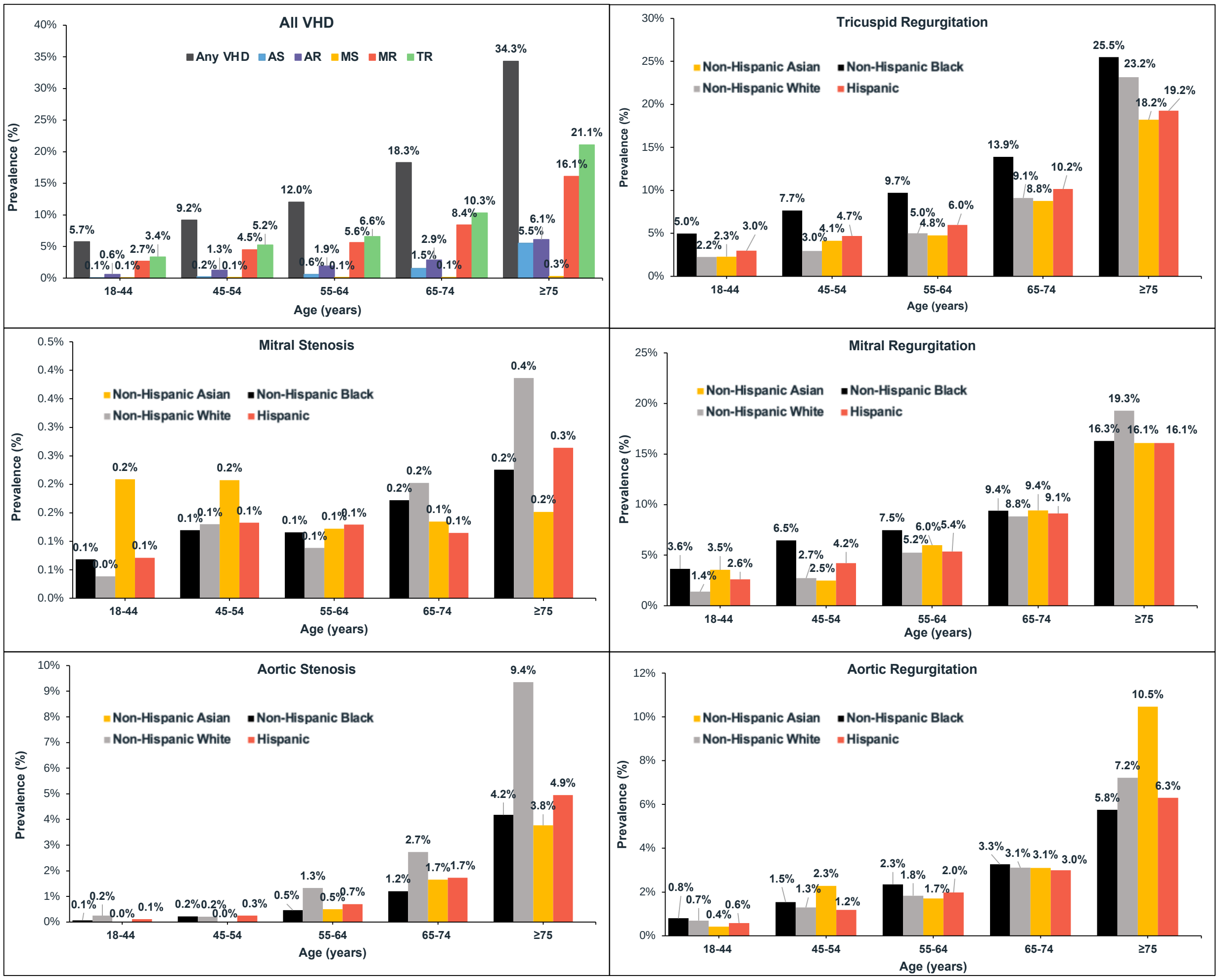

Supplement: Supplementary file 1 — Tables S1–S2 Figures S1–S4 [file JAH3-14-e035378-s001.pdf]
